# The possible dual role of Ang-2 in the prognosis of pancreatic cancer

**DOI:** 10.1038/s41598-023-45194-0

**Published:** 2023-10-31

**Authors:** Matilda Roos-Mattila, Tuomas Kaprio, Harri Mustonen, Jaana Hagström, Pipsa Saharinen, Caj Haglund, Hanna Seppänen

**Affiliations:** 1 Department of Surgery, Helsinki University Hospital, Helsinki, Finland; 2 Wihuri Research Institute, Biomedicum Helsinki, University of Helsinki, Helsinki, Finland; 3 Translational Cancer Medicine Research Program, Faculty of Medicine, University of Helsinki, Helsinki, Finland; 4 iCAN, Digital Cancer Precision Medicine, University of Helsinki and Helsinki University Hospital, Helsinki, Finland; 5 Department of Oral Pathology and Radiology, University of Turku, Turku, Finland; 6 Department of Pathology, Haartmaninkatu 3 (PB 21), University of Helsinki, 00014 Helsinki, Finland

**Keywords:** Prognostic markers, Pancreatic cancer

## Abstract

Pancreatic ductal adenocarcinoma (PDAC) features a dense desmoplastic stroma, which raises the intratumoral interstitial pressure leading to vascular collapse and hypoxia, inducing angiogenesis. Vascular growth factors, such as vascular endothelial growth factor (VEGF) and angiopoietin-2 (Ang-2), increase in PDAC. A high VEGF and a high circulating Ang-2 associate with shorter survival in PDAC. In addition to the circulatory Ang-2, PDAC endothelial and epithelial cells express Ang-2. No correlation between tumor epithelial nor endothelial cell Ang-2 expression and survival has been published. We aimed to examine Ang-2 expression and survival. This study comprised PDAC surgical patients at Helsinki University Hospital in 2000–2013. Ang-2 immunohistochemistry staining was completed on 168 PDAC patient samples. Circulating Ang-2 levels were measured using ELISA in the sera of 196 patients. Ang-2 levels were assessed against clinical data and patient outcomes. A low tumor epithelial Ang-2 expression predicted shorter disease-specific survival (DSS) compared with a high expression (*p* = 0.003). A high serum Ang-2 associated with shorter DSS compared with a low circulating Ang-2 (*p* = 0.016). Ang-2 seemingly plays a dual role in PDAC survival. Further studies are needed to determine the mechanisms causing tumor cell Ang-2 expression and its positive association with survival.

## Introduction

Pancreatic ductal adenocarcinoma (PDAC) carries the worst prognosis among the major cancers. PDAC’s absolute numbers have been steadily increasing in the western world due to the growing number of people at risk. Some researchers predict PDAC will become the second leading cause of cancer death by 2030, falling below lung cancer^1^. PDAC has a notable fibro-inflammatory component. Chronic pancreatitis is one of the best-known risk factors for PDAC^2,3^, and a high C-reactive protein (CRP) predicts poor survival better than any other currently known serum marker^4^. The fibro-inflammatory reaction leads to a dense tumor desmoplasia and a high interstitial pressure leading to vascular collapse and hypoxia^5^. Vascular growth factors are secreted in the interplay between the stromal and epithelial cells as a response to hypoxia and inflammation^6,7^.

Angiopoietins are a family of growth factors that regulate vascular remodelling and angiogenesis. Angiopoietin-1 (Ang-1) is continuously secreted from perivascular cells. By binding to its receptor Tie-2, Ang-1 maintains vascular stability. Angiopoietin-2 (Ang-2), however, has far more complex effects. Inflammation and hypoxia cause endothelial cells to express Ang-2, although, depending on the context, Ang-2 can act as a Tie-2 antagonist or weak agonist, causing vessel destabilization, endothelial cell sprouting, angiogenesis, recruitment of myeloid cells, and lymphangiogenesis^8^. In preclinical tumor models, Ang-2 stimulated tumor angiogenesis and loosened endothelial cell-to-cell junctions enhancing metastasis^9,10^.

Circulating Ang-2 increases in numerous diseases associated with inflammation and angiogenesis, such as cancer^8^. A high serum Ang-2 appears to associate with a poor prognosis in colorectal and lung cancer^11,12^, whereas a high tumor endothelial Ang-2 expression associates with a poor prognosis in some but not all cancers^13,14^. Furthermore, a high tumor stromal Ang-2 expression in non-small cell lung carcinoma associated with a better prognosis^15^.

With its strong inflammatory element, it comes as no surprise that serum Ang-2 is upregulated in PDAC as well. Shultz et al. found that a high (≥ 75th percentile) serum Ang-2 associates with a shorter survival in PDAC^16^. It is striking that Ang-2 expression was reported not only in the endothelial cells, but also in pancreatic cancer epithelial cells^16,17^. While a high circulating Ang-2 level has been associated with a poorer prognosis, no association between tumor epithelial nor endothelial Ang-2 expression and survival in PDAC has been published thus far.

This study aimed to investigate the effect of Ang-2 protein in survival. We did so by comparing the associaton between circulating Ang-2 using enzyme-linked immunosorbent assay (ELISA) and the pancreatic tumor expression of Ang-2 using immunohistochemistry in both the cancer cell and endothelial expressions of Ang-2.

## Results

### Patient samples

The study setting consisted of 216 patients operated on at Helsinki University Hospital between 2000 and 2013. Tissue for immunohistochemistry was available from 168 patients and serum from 196 patients. We only included patients with pancreatic ductal adenocarcinoma without neoadjuvant therapy in the study. The median age at surgery was 65 years (range 39–84). Clinical data were obtained from patient records and survival data were provided by the Finnish Population Registration Center and Statistics Finland.

### Immunohistochemical staining

**Figure 1:**
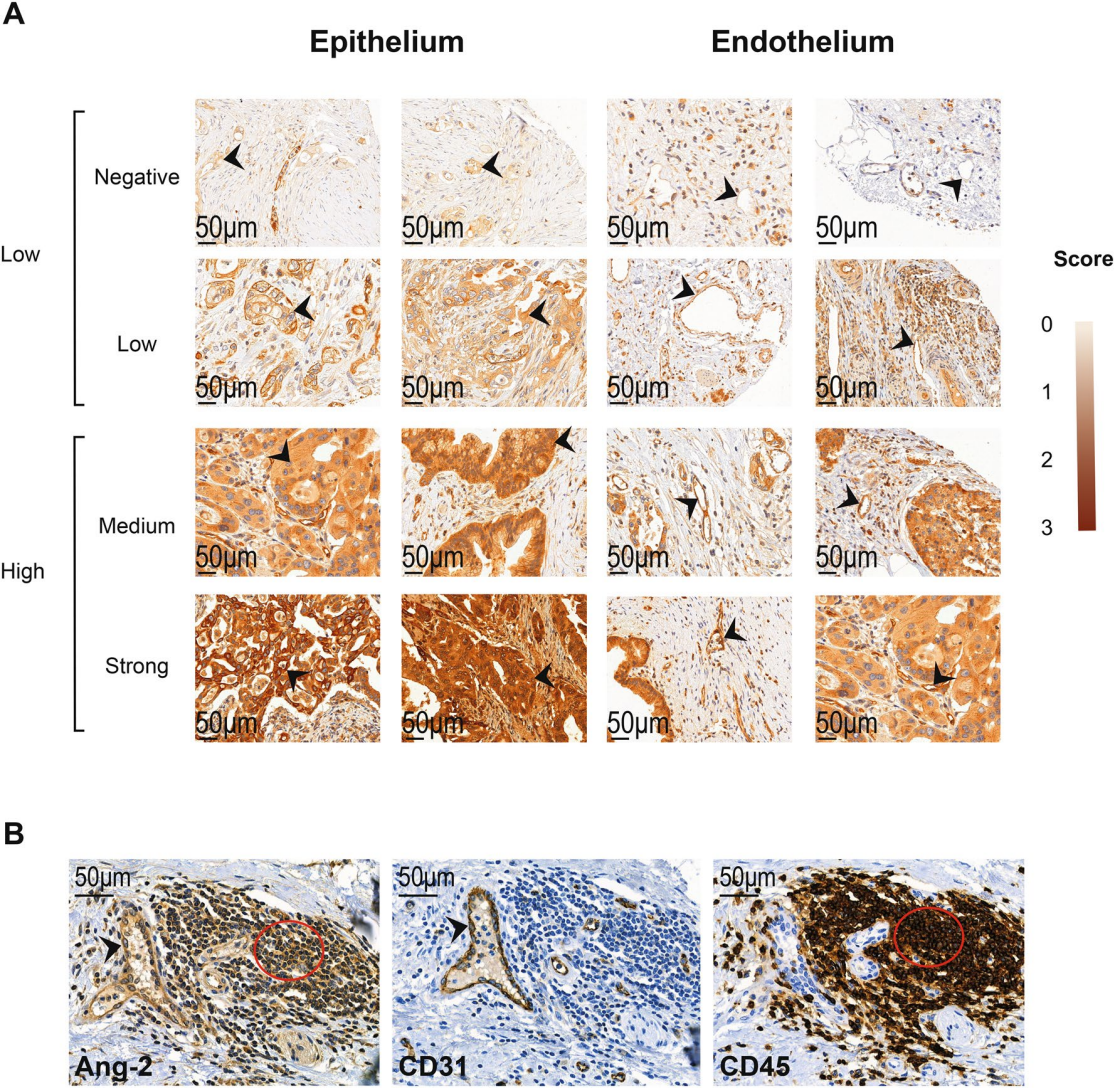
Both tumor epithelial and endothelial cells, as well as lymphocytes stained positive for Ang-2. (**A**) Tumor epithelial (black arrows in left column) and endothelial (black arrows in right column) staining of Ang-2 with staining intensity ranging from negative (0) to strong (3). (**B**) Double stainings of consecutive tissue slides show Ang-2 expression in blood vessel coating endothelial cells (CD31, black arrow head) and lymphocytes (CD45, red circle).

Ang-2 was expressed both in the cytoplasm of pancreatic cancer epithelial cells and in the endothelial cells (Fig. 1A).

### Tumor epithelial Ang-2 expression

Of the 168 tissue microarray (TMA) samples, 158 (94.0%) contained sufficient representative tumor tissue to allow for the evaluation of Ang-2 epithelial staining (Fig. 1A). Nearly half of the patients (77 patients, 48.7%) showed a moderate expression of Ang-2. Scores were dichotomized based on their Ang-2 expression. Negative to low scores (< 2) were categorized as low expression and moderate to strong (≥ 2) scores were categorized as high expression (Fig. 1A). Only two out of the 158 stained tumors were completely Ang-2 negative in their epithelium (1.3%). An additional two tumors had one section with negative Ang-2 expression, but expressed Ang-2 in other parts of the tumor epithelium (see Supplementary Table 1).

### Association between tumor epithelial Ang-2 expression and clinicopathological variables

The intensity of the tumor epithelial Ang-2 expression was not significantly associated with age, sex, stage, lymph node metastasis, grade, tumor size, perineural, or perivascular infiltration (Table 1).

**Table 1:** Association between tumor (epithelial and endothelial) and serum Ang-2 expressions and clinicopathological parameters.

	Epithelial			Endothelial			Serum		
	Low	High	*p*	Low	High	*p*	Low	High	*p*
N (%)	59 (37.3)	99 (62.7)		61 (38.4)	98 (61.6)		109 (55)	87 (45)	
Age									
< 65	28 (47)	52 (53)	0.62	28 (46)	53 (54)	0.33	54 (49)	40 (46)	0.67
≥ 65	31 (53)	47 (47)		33 (54)	45 (46)		55 (51)	47 (54)	
Sex									
Female	24 (41)	47 (47)	0.42	24 (39)	47 (48)	0.32	43 (39)	41 (47)	0.31
Male	35 (59)	52 (53)		37 (61)	51 (52)		66 (61)	46 (53)	
Stage									
IA–IIA	13 (24)	28 (29)	0.57	15 (25)	26 (27)	0.85	35 (33)	21 (25)	0.27
IIB–IV	42 (76)	70 (71)		44 (75)	69 (73)		72 (67)	63 (75)	
Missing	4	2		2	3		2	3	
Lymph node ratio									
< 20%	43 (74)	76 (78)	0.56	51(84)	68 (72)	0.12	89 (83)	54 (67)	0.01
≥ 20%	15 (26)	21 (22)		10 (26)	27 (28)		18 (17)	27 (33)	
Missing	1	2		0	3		2	6	
Grade									
1	5 (10)	11 (12)	0.90	5 (9)	11 (12)	0.42	17 (17)	7 (10)	0.32
2	38 (73)	64 (70)		42 (79)	61 (66)		64 (64)	54 (73)	
3	9 (17)	17 (18)		6 (11)	20 (22)		19 (19)	13 (18)	
Missing	7	7		8	6		9	13	
Tumor size									
< 3 cm	24 (41)	61 (56)	0.25	27 (44)	47 (49)	0.62	55 (51)	37 (46)	0.46
≥ 3 cm	34 (59)	47 (44)		34 (56)	48 (51)		52 (49)	44 (54)	
Missing	1	2		0	3		2	6	
Perineural infiltration									
No	13 (26)	18 (26)	0.95	14 (29)	18 (25)	0.83	27 (30)	12 (19)	0.19
Yes	37 (74)	50 (74)		35 (71)	52 (74)		63 (70)	50 (81)	
Missing	9	31		12	28		19	25	
Perivascular infiltration									
No	33 (67)	41 (63)	0.64	27 (59)	48 (70)	0.32	61 (72)	32 (58)	0.10
Yes	16 (33)	24 (37)		19 (41)	21 (30)		24 (28)	23 (42)	
Missing	10	34		15	29		24	31	

The epithelial Ang-2 intensity negatively correlated with carbohydrate antigen 19-9 (CA19-9) (*p* = 0.001, Spearman’s rho − 0.261). There was no statistically significant correlation between epithelial Ang-2 expression with either CRP or carcinoembryonic antigen (CEA) (*p* = 0.958, Spearman’s rho -0.005 and *p* = 0.227, Spearman’s rho − 0.099).

### Endothelial Ang-2 expression

Of the 168 TMA samples, 159 (94.6%) contained sufficient representative tissue to allow for the evaluation of the Ang-2 endothelial staining. Ang-2 was expressed in the endothelial cells of pancreatic tumors (Fig. 1A). To confirm the cell type as endothelial cells, we performed double staining of consecutive slides using endothelial marker CD31 (Fig. 1B).

In accordance with the tumor Ang-2 expression, nearly half of the patients (73 patients, 45.9%) showed a moderate endothelial expression of Ang-2 (Table 2). We dichotomized the scores with negative to low scores (< 2) as a low expression and moderate to strong (≥ 2) scores were categorized as a high expression (Fig. 1A). None of the patients had a negative Ang-2 expression in their endothelium (Supplementary Table 2).

**Table 2:** Cox univariable analysis of relative risk of death from pancreatic ductal adenocarcinoma.

	Hazard ratio (95% CI)	*p* value
Age*	1.01 (0.99–1.03)	0.19
Sex (male)	1.07 (0.80–1.43)	0.64
Stage IA–IIA	1.00	
Stage IIB	1.51 (1.05–2.17)	0.025
Stage III–IV	2.61 (1.77–3.83)	< 0.001
Grade 1	1.00	
Grade 2	0.98 (0.64–1.50)	0.92
Grade 3	1.74 (1.03–2.95)	0.038
Adjuvant treatment (yes)	0.77 (0.58–1.03)	0.077
Radical operation	0.59 (0.40–0.86)	0.003
Ang-2 epithelial expression (high)	0.59 (0.42–0.84)	0.003
Ang-2 serum (high)	1.45 (1.07–1.97)	0.016
Ang-2 endothelial expression (high)	0.93 (0.66–1.30)	0.66
logCA19-9*	1.26 (1.08–1.46)	0.003
logCRP*	1.63 (1.21–2.18)	0.001
logCEA*	1.68 (1.07–2.66)	0.026

*Analyzed as continuous logarithmic (base 10) transformed variable.

**Figure 2:**
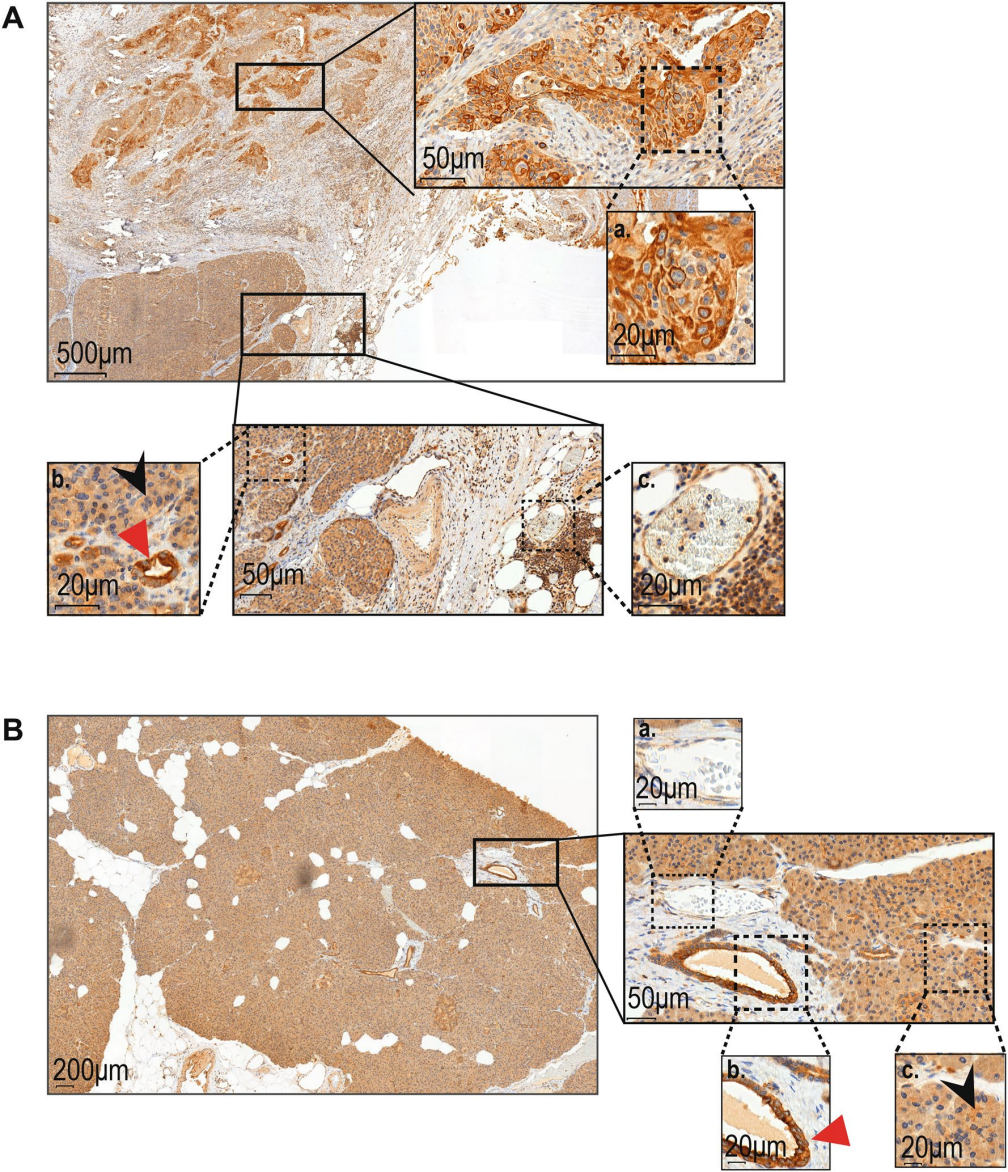
Histologically normal pancreatic tissue stains positive for Ang-2. Whole block slides of (**A**). PDAC tissue (a.) with adjacent ductal and acinar (b.) structures of the normal pancreas. Endothelial cells of a vessel with surrounding lymphocytes are shown in c. (**B**) normal pancreatic tissue adjacent to neuroendocrine tumor with endothelial (a.), ductal (b.) and acinar (c.) cells staining positive for Ang-2. Black arrows points at acinar structures and red arrow at ductal.

In addition to the previously described tumor epithelial and endothelial, we noticed in a small proportion of tumors the presence of a population of Ang-2 expressing lymphocyte-like cells (Fig. 2Ac.), which were confirmed as lymphocytes in double staining using common lymphocyte marker CD45 (Fig. 1B).

### Association between endothelial Ang-2 expression and clinicopathological variables

The intensity of the endothelial Ang-2 expression did not significantly associate with age, sex, stage, lymph node metastasis, grade, tumor size, perineural, or perivascular infiltration (Table 1).

In contrast to the epithelial Ang-2 expression, endothelial Ang-2 intensity correlated positively with CA19-9 (*p* = 0.012, Spearman’s rho 0.218). There was no statistically significant correlation with either CRP or CEA (*p* = 0.349, Spearman’s rho − 0.166 and *p* = 0.160, Spearman’s rho 0.124). The tumor epithelial and endothelial expressions of Ang-2 showed a positive correlation (*p* = 0.017, Spearman’s rho 0.240).

### Ang-2 expression in histologially benign pancreatic tissue

We found that Ang-2 expression was not limited to the tumor tissue alone (Fig. 2A). To investigate this further we stained PDAC whole block slides with Ang-2 (n = 5). We found that in addition to the PDAC tumor tissue (Fig. 2Aa), histologically benign pancreatic ductal and acinar (Fig. 2Ab.) tissue also showed positive Ang-2 staining. To determine whether it was due to the closely located PDAC (and subsequent inflammation), we stained whole block slides from neuroendocrine tumors with Ang-2 (n = 5) (Fig. 2B). Again, histologically benign pancreatic ductal (Fig. 2Bb.) and acinar (Fig. 2Bc.) tissue stained positive for Ang-2. Also endothelial cells of vessels within the histologically normal pancreatic tissue (Fig. 2Ac., Bd.) stained positive for Ang-2.

### ELISA

The optimal cut-off of 2.83 ng/ml for serum Ang-2 was found through a time-dependent receiver operating curve (ROC) analysis. The median circulating Ang-2 level in patients with PDAC was 2.72 (range 0.36–26.88). Only three patients had Ang-2 expression levels exceeding 10 ng/ml. We found no significant correlation between the serum Ang-2 and the Ang-2 level in the tumor epithelial (*p* = 0.330, Spearman’s rho − 0.079) or endothelial (*p* = 0.600, Spearman’s rho − 0.041) immunohistochemistry (IHC) staining.

### Association between serum Ang-2 expression and clinicopathological variables

A high lymph node ratio (ratio above 20%) was more common in patients with higher levels of circulating Ang-2 (33% versus 17%, *p* = 0.01). The level of circulating Ang-2 did not significantly associate with age, sex, tumor stage, grade, size, number of lymph node metastasis, perivascular infiltration, or perineural infiltration (Table 1).

The serum Ang-2 expression correlated positively with CEA (*p* = 0.005, Spearman’s rho 0.205) and CRP (*p* < 0.001, Spearman’s rho 0.286). The circulating Ang-2 showed no statistically significant correlation with CA19-9 (*p* = 0.106, Spearman’s rho 0.118).

Schultz et al.^9^ previously reported that a serum Ang-2 exceeding the 75th percentile associated with a higher degree of lymph node metastasis. While in our series the serum Ang-2 did not significantly associate with the lymph node metastasis ratio directly, we separately examined high versus low serum Ang-2 expressions. We found a positive correlation between a > 20% lymph node ratio (LNR) and serum Ang-2 levels exceeding the 50th (*p* = 0.41, Spearman’s rho 0.149) and 75th (*p* = 0.005, Spearman’s rho 0.203) percentile. An LNR < 20% was found in 19% versus 39.1% of patients with lower than versus higher than 75th percentile serum Ang-2 expression levels (*p* = 0.010).

### Survival analysis

**Figure 3:**
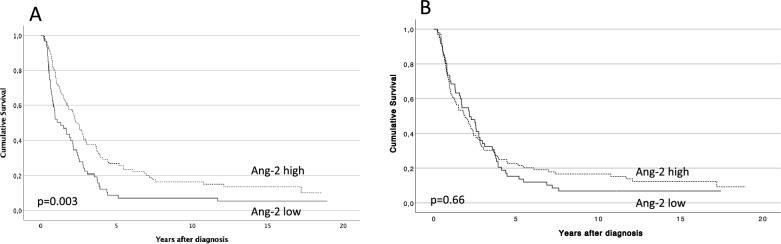
Impact of Ang-2 expression on disease-specific survival using the Kaplan–Meier analysis. (**A**) Epithelial expression and (**B**) endothelial expression. Log-rank test was used.

In a univariate analysis, a stronger Ang-2 tumor epithelial expression associated with a better prognosis (lower hazard ratio [HR]; Table 2). Furthermore, this result persisted in a multivariate analysis (low versus high Ang-2 expression; HR = 0.49; 95% confidence interval [CI] 0.31–0.78; *p* = 0.003) adjusted for age, sex, stage, adjuvant therapy, radical (R0) surgery, and tumor markers CA19-9, CRP, and CEA (Table 3). We detected a shorter disease specific survival (DSS) in patients with a low tumor epithelial Ang-2 expression (median 1.33; 95% CI 0.50–2.16) when compared with those with an intermediate to high expression (median 2.40; 95% CI 1.86–2.94; *p* = 0.003; Fig. 3A**)**.

**Table 3:** Cox multivariable analysis of relative risk of death from pancreatic ductal adenocarcinoma.

Multivariable—Ang-2 tumor expression		
	Hazard ratio (95% CI)	p value
Age (years)	1.00 (0.98–1.03)	0.91
Sex (male)	0.97 (0.63–1.50)	0.89
Stage IA–IIA	1.00	
Stage IIB	2.23 (1.23–4.04)	0.008
Stage III–IV	3.74 (1.96–7.15)	< 0.001
Adjuvant treatment (yes)	0.71 (0.47–1.08)	0.11
Radical surgery (R0)	0.57 (0.34–0.95)	0.03
Ang-2 epithelial expression (high)	0.49 (0.31–0.78)	0.003
logCA19-9	1.27 (0.99–1.63)	0.06
logCRP	1.48 (1.02–2.14)	0.04
logCEA	1.33 (0.64–2.77)	0.44

Multivariable—Ang-2 serum expression		
	Hazard ratio (95% CI)	*p* value
Age	1.01 (0.99–1.03)	0.40
Sex (male)	1.02 (0.74–1.31)	0.91
Stage IA–IIA	1.00	
Stage IIB	1.96 (1.29–2.96)	0.001
Stage III–IV	2.75 (2.05–5.04)	< 0.001
Adjuvant therapy administered	0.63 (0.45–0.88)	0.006
Radical surgery	0.71 (0.48–1.06)	0.09
Serum Ang-2 (high)	1.48 (1.08–2.04)	0.02

No statistically significant association was found between the endothelial Ang-2 expression and prognosis in univariate analysis (*p* = 0.66; Table 2; Fig. 3B).

**Figure 4:**
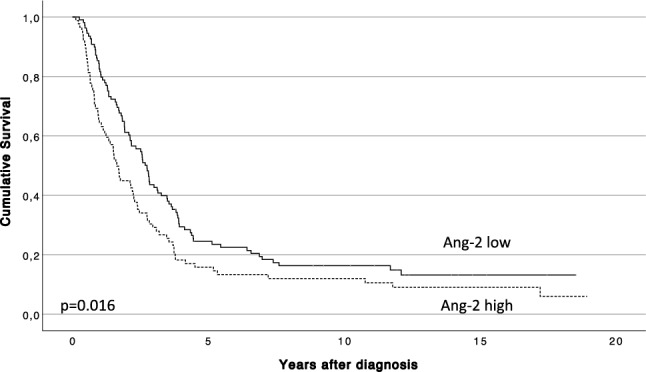
Impact of Ang-2 serum levels on disease-specific survival using the Kaplan–Meier analysis. Log-rank test was used.

A high serum Ang-2 expression associated with a poorer prognosis according to a univariable analysis (*p* = 0.016; HR = 1.45; 95% CI 1.07–1.97; Table 2). A multivariable analysis (Ang-2 expression: HR = 1.48; 95% CI 1.08–2.04; *p* = 0.015), adjusted for age, sex, stage, adjuvant therapy, and radical (R0) surgery, was significant. Yet, when CA19-9, CRP, and CEA were added to the model, the multivariable analysis was not significant (*p* = 0.081). In addition, a high serum Ang-2 expression associated with a shorter DSS (median 1.63; 95% CI 1.37–1.89 years) when compared with patients with a lower serum Ang-2 expression (median 2.74; 95% CI 2.33–3.16; *p* = 0.016; Fig. 4).

## Discussion

In this study, we found that tumor epithelial Ang-2 expression strongly and significantly associates with PDAC survival whereby a higher tumor expression of Ang-2 predicts a better survival. By contrast, a higher serum Ang-2 level predicts a poorer survival.

In concordance with our findings, one previous study has found higher serum Ang-2 levels to be associated with poorer prognosis (and a higher number of lymph node metastases in PDAC)^9^. However, in that study, the association was found in serum Ang-2 levels over the 75th percentile. In our patient samples series, the levels of serum Ang-2 were generally lower than those reported by Schulz et al., and neither the 75th percentile of our sample nor the cut-off used in the Schultz et al. study predicted survival in our cohort^9^. The ratio of positive lymph node metastases was, however, higher in patients with a higher than 50th and 75th percentile serum Ang-2, which agrees with Schultz et al.’s findings.

The association between serum Ang-2 and tumor (epithelial and endothelial) Ang-2 expression was inverse in our study, as discussed later. This inverse relationship suggests that the serum Ang-2 is secreted from sources outside the tumor; most probably from the systemic vasculature. The exact origin of the serum Ang-2 can, however, not be answered by the means of this study. Alternatively, epithelial Ang2 staining might in theory represent Ang2 bound to a cell surface receptor in epithelial cells.

Schultz et al. found that Ang-2 was transcriptionally active only in transformed ductal cells, but not in the cells of a normal pancreas^9^. Indeed, Ang-2 expression is known to be enhanced in KRAS mutated cancers through transcriptional upregulation mediated by FOXC2 transcription factor^18^. In our study, however, also histologically normal pancreatic epithelium expressed Ang-2. Further, in the Human Protein Atlas, the glandular cells in a normal exocrine pancreas express Ang-2 at moderate levels^19^.

While previous studies have confirmed that Ang-2 is expressed in the epithelial cells of PDAC tumors, to our knowledge this is the first time the association between Ang-2 expression and survival has been examined. Surprisingly, we found that the relationship between tumor epithelial Ang-2 and survival was opposite of that of serum Ang-2 and survival.

Commercial pancreatic cell lines such as Mia Paca-2, PANC-1, and AsPC-1 have been shown to express Ang-2 mRNA^9,20^, and their aggressiveness is associated, and can be induced by upregulation of Ang-2^9,20^. Further, downregulation of Ang-2 has been shown to suppress invasion and colony formation of pancreatic cell lines in vitro^20^*.* In addition, both Ang-2 silencing in pancreatic cancer cells and intratumoral injection of miR-145 (causing downregulation of presumably both tumor epithelial and endothelial Ang-2) slowed the growth of pancreatic xenografts in nude mice^20–22^. Based on our results, it seems that the effect of tumor epithelial Ang-2 on prognosis is, however, far more complicated in PDAC patients than suggested by preclinical models. Indeed, albeit promising preclinical results, Ang-2 inhibitors have showed limited effect in clinical trials^23,24^.

It needs to be noted that in addition to of the Ang-2 Tie-2 signaling pathway, Ang-2 is also known activate integrins signaling^25,26^. The integrin signaling of Ang-2 is known to be involved in the autocrine signaling between PDAC epithelial cells^9^ and regulate tumor matrix metalloproteinase (MMP) expression^27,28^. Clinical trials modifying tumor extracellular matrix in PDAC, such as the HALO-trial, have taught us that the results of such modifications are not always straightforward^29^.

In addition, Ang-2 is known to affect the immune cell environment of the tumor^30,31^. The most promising results from Ang-2 inhibitors have been gained from combination treatment with either immunotherapy or other anti-angiogenic drugs^9^. Whereas many of the prior studies have been conducted in immunodeficient mice, Schmittnaegel et al. showed that in immunocompetent models combined Ang2 and VEGF blocking antibody therapy relied on cytotoxic T cells^32^. Further research is needed to explore the role of immune cells in the prognosis associated with Ang-2 expression.Overall, the underlying mechanism (and cellular components involved) leading to the better prognosis in Ang-2 expressing tumor epithelial cells is of importance and should be addressed in future studies. Most importantly, this should be investigated before considering the use of anti-Ang-2 therapies in PDAC patients.

The endothelial Ang-2 expression did not, in our study, correlate with PDAC survival. Immunohistochemical analysis of endothelial Ang2 in other cancer types has shown variable correlation with survival, for example^13^ showed that high endothelial Ang2 in RCC correlated with better prognosis, whereas^33^ showed that high Ang2 correlated with poor prognosis in glioblastoma. Nasarre et al. have showed using preclinical models that endothelial Ang-2 is vital during the early stages of tumorigenesis, and both sarcoma and melanoma tumor cells grew slower into tumors in Ang-2 deficient mice. However, the tumor growth during later stages were unchanged between Ang-2 deficient and wild type mice^34^. The blockade of Ang-2 can also induce vascular normalization, rather than vascular depletion^8^. The balance of decreasing delivery of nutrients and thereby inhibiting tumor growth—but not impairing the routes cytostatic drugs to enter the tumor is, again, not straighforward.

Regardless of the underlying mechanism, our findings suggest that the local inflammatory (tumor Ang-2 expression) state associates with a better prognosis, while the systemic inflammation (serum Ang-2) associates with a poorer prognosis. This agrees with previous findings in PDAC as well as in other cancers whereby local inflammatory responses appear beneficial to patient survival, while systemic inflammation is disadvantageous^35^.

Another interesting finding from this study was the cytosolic Ang-2 expression in intratumoral lymphocytes. Monocytes and macrophages are known to express Tie-2. To the best of our knowledge, the expression of Ang-2 within lymphocytes has not previously been described in the literature. However, many of the stromal cells reportedly expressing Ang-2 are lymphocytes, based on images in an article by Andersen et al.^15^. In addition, the Single Cell Expression Atlas shows Ang-2 expressing lymphocytes in glioblastoma^36^, while chronic lymphocytic leukemia cells also show immunoreactivity to Ang-2^37^. The Human Protein Atlas also shows Ang-2 expression in immune cells, such as basophils^19^. Perhaps, the cytosolic Ang-2 staining in fact results from its expression within lymphocytes. However, further studies are required to confirm this supposition.

The strength of this study lies in the large cohorts we analyzed with long follow-up times and reliable clinical data. Since patients were recruited to the study when awaiting pancreatic surgery, the number of stage IV patients remained rather low in our series (n = 8). This does not reflect the general PDAC population, > 80% of whom present with inoperable tumors. Further studies are needed to determine how high serum Ang-2 levels are in widely metastasized PDAC and how it predicts survival in such patients. It would also be interesting to determine whether Ang-2 expression is detectable in the metastatic sites of PDAC, given that histological samples from PDAC metastases are rarely available. The optimal cut-off for serum Ang-2 was found through a time-dependent ROC analysis. Using this cut-off, the Kaplan–Meier as well as the Cox regression analyses were significant, as was the multivariable model. However, the serum Ang-2 results should be interpreted carefully, since this cut-off value has not been employed in a different cohort.

In summary, we identify here an interesting potential dual role of Ang-2 in PDAC: the local beneficial effect of tumor epithelial Ang-2 and the role of serum Ang-2 as a predictor of a poor prognosis. Further studies are needed to identify the mechanisms causing tumor epithelial Ang-2 and normal pancreatic expression, along with the positive association with survival.

## Methods

### Ethical approval

The Surgical Ethics Committee of Helsinki University Hospital (Dnro HUS 226/E6/06, extension TMK02 §66 17.4.2013) and the National Supervisory Authority of Health and Welfare (Valvira Dnro 10041/06.01.03.01/2012) approved the study. Patients provided their written informed consent upon inclusion in the study Patient information, samples and data were handled and stored in accordance with the Declaration of Helsinki and other local regulations.

### Preparation of tumor tissue microarrays and immunohistochemistry

We collected paraffin-embedded surgical samples from the archives of the Department of Pathology, Helsinki University Hospital. The histopathological diagnosis of PDAC was re-evaluated by two experienced pathologists. Representative regions of a tumor were marked on haematoxylin & eosin-stained slides. Tissue microarray blocks were prepared and six 1.0-mm punches from each tumor sample were cut using a semiautomatic tissue microarrayer (Tissue Arrayer 1, Beecher Instruments Inc., Silver Spring, MD, USA). TMA blocks were cut into 4-μm sections, deparaffinized, rehydrated, and incubated in a Tris–HCl buffer at 98 ºC for 20 min. TMA slides were stained in an Autostainer 480 (Lab Vision Corp., Fremont, CA, USA) using the Dako REAL EnVision Detection system. We used goat polyclonal antibodies to human Angiopoietin-2 (Serial number AF623, amino acid residues Asp68-Phe496, 1:800, 0.2 mg/ml, R&D Systems, Minneapolis, MN, USA). Samples of colon tissue and normal lymph nodes served as positive controls in each staining series. To increase the representativeness, a total of six spots were examined per patient. These spots were from different areas of a PDAC tumor.

Immunohistochemistry staining was conducted on 159 PDAC patient samples. We chose five whole PDAC tissue blocks to evaluate Ang-2 expression in tumor-adjacent tissue. Double staining of Ang-2 together with endothelial marker CD31 and common lymphocyte marker CD45 were done on four of these whole tissue blocks to confirm the cell types of Ang-2 expressing cells. An additional five sections of a normal pancreas were obtained from surgeries removing local pancreatic neuroendocrine tumors.

### Evaluation of staining

Cytoplasmic staining of Ang-2 in both pancreatic tumor cells as well as tumor endothelial cells were blindly scored by two examiners (MJ and JH), the second of whom is an experienced pathologist. The staining intensity was categorized as negative (0), weakly positive (1), moderately positive (2), and strongly positive (3) (Fig. 1A). The investigators were blinded to each other’s scores as well as to the clinical data. In the event that the intensity was scored differently between investigators, a consensus score was determined through discussion. A median score for each sample was calculated.

### ELISA

ELISA were performed on 196 patient serum samples. The assay was performed on an MTX LabSystems Multiskan EX reader (MTX LabSystems, Bradenton, FL, USA). We used the DuoSet methodology ELISA (DANG20, R&D Systems, Biotechne, Minneapolis, MN, USA) assay to measure circulating Ang-2 levels in patient sera. Samples were quantified by converting optical density values to concentrations (pg/ml) using a standard curve calculated in accordance with the manufacturer’s instructions. In this assay, the quantification limit was 46.9 pg/ml and the detection limit was 8.29 pg/mL. None of the samples showed concentrations lower than the quantification or detection limits. This method was described in greater detail elsewhere^38^. The optimal cut-off point was selected based on the Youden index and set to 2.83 ng/ml. Time-dependent ROC curve is shown in Supplementary Fig. 1.

### Statistical analysis

Associations between Ang-2 and clinical data and patient characteristics were assessed using the Fisher’s exact test or the Cochran–Armitage test. Descriptive survival analysis was performed using the Kaplan–Meier method and groups were compared using the log-rank test. DSS was calculated from the day of surgery until death from PDAC or until the end of the follow-up period. The time-dependent receiver operating curve (ROC) was constructed for DSS at one year follow-up and the cut-off value for the serum Ang-2 was obtained by maximizing the Youden index, granting equal weight to sensitivity and specificity. Uni- and multivariable survival analyses were calculated using the Cox regression analysis. The Cox regression assumption of a constant hazard ratio over time was assessed by plotting the Schoenfeld residuals over time, and testing for a trend. No significant deviations from the proportional hazards assumptions were identified. Interactions were considered in the multivariable models, but no statistically significant interactions were detected after applying the Bonferroni correction for multiple testing. The non-parametric Spearman’s correlation coefficient (rho) was used to assess correlations between variables. We considered *p* ≤ 0.05 as stastistically significant, and all tests were two-sided. All statistical analyses were performed using SPSS version 27.0 (IBM SPSS Statistics version 27.0 for Mac; SPSS Inc., Chicago, IL, USA, an IBM Company) and R version 4.0.3 (Foundation for Statistical Computing, Vienna, Austria).

### Supplementary Information

Supplementary Information 1.

Supplementary Information 2.

## Data Availability

The datasets used and/or analysed during the current study are available from the corresponding author on reasonable request.
