# Supplementary figures and images for: The possible dual role of Ang-2 in the prognosis of pancreatic cancer
Source: Sci Rep. 2023 Oct 31;13:18725. doi: 10.1038/s41598-023-45194-0 (PMC10618172; doi:10.1038/s41598-023-45194-0)

# Time dependent AUC at 7 months

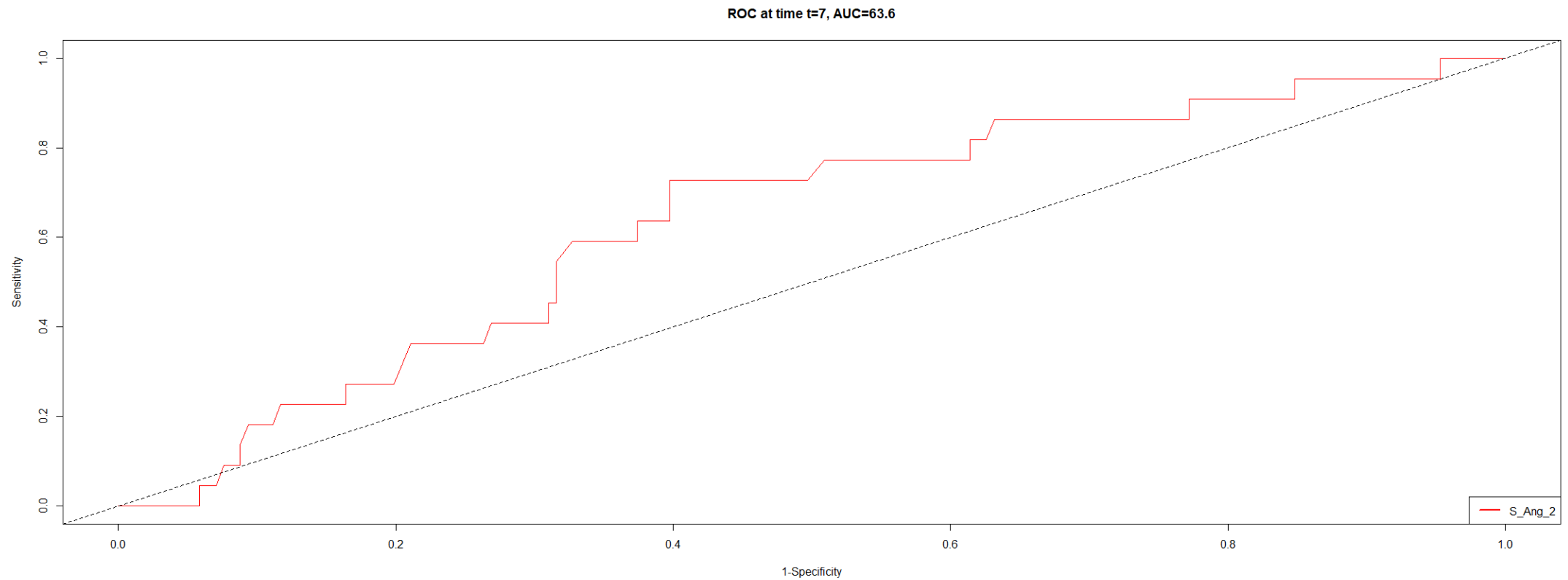

Supplement: Supplementary file 1 — Supplementary Information 1. [file 41598_2023_45194_MOESM1_ESM.pdf]
