# Supplementary material for: The possible dual role of Ang-2 in the prognosis of pancreatic cancer
Source: Sci Rep. 2023 Oct 31;13:18725. doi: 10.1038/s41598-023-45194-0 (PMC10618172; doi:10.1038/s41598-023-45194-0)
Supplement: Supplementary file 2 — Supplementary Information 2. [file 41598_2023_45194_MOESM2_ESM.docx]

SupplementaL Materials:

Supplementary Table 1. Proportions of tumor ang-2 staining scores

| **Median score** | **N (%)** | **Score category** | **N (%)** |
| --- | --- | --- | --- |
| 0 | 2 (1.3) | Low, <2 | 59 (37.3) |
| 0.5 | 0 (0) |  |  |
| 1 | 35 (22.2) |  |  |
| 1.5 | 22 (13.9) |  |  |
| 2 | 77 (48.7) | High, ≥2 | 99 (62.7) |
| 2.5 | 10 (6.3) |  |  |
| 3 | 12 (7.6) |  |  |
| Total | 158 (100) |  |  |

Supplementary Table 2. Proportions of endothelial ang-2 staining scores

| **Median score** | **N (%)** | **Score category** | **N (%)** |
| --- | --- | --- | --- |
| 0 | 0 (0) | Low, <2 | 61 (38.4) |
| 0.5 | 0 (0) |  |  |
| 1 | 40 (25.2) |  |  |
| 1.5 | 21 (13.2) |  |  |
| 2 | 73 (45.9) | High, ≥2 | 98 (61.6) |
| 2.5 | 10 (6.3) |  |  |
| 3 | 15 (9.4) |  |  |
| Total | 159 (100) |  |  |
